# Belzutifan-induced tumor regression in sporadic hemangioblastoma: a case report and literature review

**DOI:** 10.1007/s11060-026-05466-x

**Published:** 2026-02-14

**Authors:** Rebekka E. Hooks, Niket Yadav, Mark Willy L. Mondia, Georgios Mantziaris, Alaa Saleh, Anna Vi Jones, Matthew McCord, Melike Mut, Ashok R. Asthagiri, Benjamin W. Purow

**Affiliations:** 1 Division of Neuro-Oncology, Department of Neurology, University of Virginia, Charlottesville, Virginia USA; 2 Medical Scientist Training Program (MSTP), University of Virginia, Charlottesville, Virginia USA; 3 Division of Adult Neurology, Department of Neurosciences, Philippine General Hospital, Manila, Philippines; 4 Department of Neurosurgery, University of Virginia, Charlottesville, Virginia USA; 5 Department of Pathology, University of Virginia, Charlottesville, Virginia USA; 6 Department of Radiology and Medical Imaging, University of Virginia, Charlottesville, Virginia USA; 7 University of Virginia, 21 Hospital Drive, Old Medical School, Room 4881, Charlottesville, VA 22908 USA

**Keywords:** Sporadic hemangioblastoma, CNS, Belzutifan, VHL, HIF-2α

## Abstract

**Background:**

Sporadic hemangioblastomas (sHBs) are less prevalent than those in von Hippel-Lindau (VHL) disease. Surgical resection and radiation therapy remain the standard treatments, and medical treatment options for sHBs are limited. While belzutifan shows a favorable response in VHL-associated HBs, its efficacy in sHBs is uncertain. We present a case of probable belzutifan-induced tumor reduction in a patient with sHB.

**Methods and results:**

We present a case of a 65-year-old man with progressive right-sided hemifacial paresthesia, retro-orbital pain, and periorbital edema, subsequently diagnosed with progressive right trigeminal nerve-associated sHB. The patient did not meet clinical or genetic criteria for VHL disease. Serial magnetic resonance imaging (MRI) demonstrated significant tumor progression over seven years. Treatment included 12.5 Gy of radiation in 2016, subtotal resection in 2021, and re-radiation with 50.6 Gy in 2022. Repeat debulking resection was deemed high-risk due to tumor location, and prior treatment precluded further re-radiation. Following multidisciplinary review and patient preference, medical therapy was pursued, and belzutifan at 120 mg/day was initiated. MRI demonstrated tumor reduction from 3.4 cm to 3.1 cm by month 2. Due to anemia and fatigue, belzutifan was reduced and continued at 80 mg/day with stable hemoglobin above 10.0 and tolerable fatigue. Serial brain MRIs showed a durable tumor reduction, with a −7.00 mm/year median linear growth rate reduction by month 13.

**Conclusion:**

To our knowledge, this is the first report of belzutifan treatment for sHB, resulting in tumor reduction. Belzutifan may offer a possible treatment option for residual, progressive, and/or pre-surgical sHBs.

## Introduction

Hemangioblastomas (HBs) are classified as World Health Organization (WHO) grade 1 tumors, predominantly occurring within the central nervous system (CNS), and constitute approximately 1–2% of intracranial CNS tumors and up to 10% of spinal cord tumors [1, 2]. These rare, vascular-rich, histologically benign neoplasms that originate from mesoderm-derived stromal cells, and may present sporadically (55–75%) or in association with VHL disease, an autosomal-dominant disorder characterized by multi-organ tumor susceptibility [1, 3]. In the context of VHL disease, CNS hemangioblastomas frequently manifest as multiple lesions, whereas solitary occurrences are more typical in sporadic cases [1]. Anatomically, CNS hemangioblastomas have a predilection for the cerebellum (45–79.8%) and the spinal cord (30–45%), with lower affinity for the brainstem (5–16.9%) [4–6]. Supratentorial HBs, although less common, account for roughly 27% of all cranial HBs, including sporadic and VHL-associated cases, and are most commonly located in the frontal and sellar/parasellar regions [5–8]. Cranial nerve involvement is exceedingly rare (<1%), most often affecting the optic nerve [9, 10].

Management is challenging due to cyst formation, hypervascularity, and growth heterogeneity, often characterized by saltatory growth followed by exponential/linear growth [5, 8]. Surgical gross total resection (GTR) remains the main therapeutic option for cranial HBs. Stereotactic radiosurgery (SRS) is an alternative option, particularly for inaccessible tumors or for residual/recurrent disease, demonstrating favorable 5- and 10-year tumor control rates in the absence of effective systemic therapies [7]. Anti-angiogenic (e.g., bevacizumab, pazopanib, anlotinib) and apoptosis-promoting drugs (e.g., somatostatin analogs) show potential promise as emerging therapies for HB in case reports [11]. Studies have explored hypoxia-inducible factor (HIF) targeted therapy for HB treatment, specifically the selective inhibition of HIF-2α given its role as a downstream mediator of VHL loss.

**Fig. 1:**
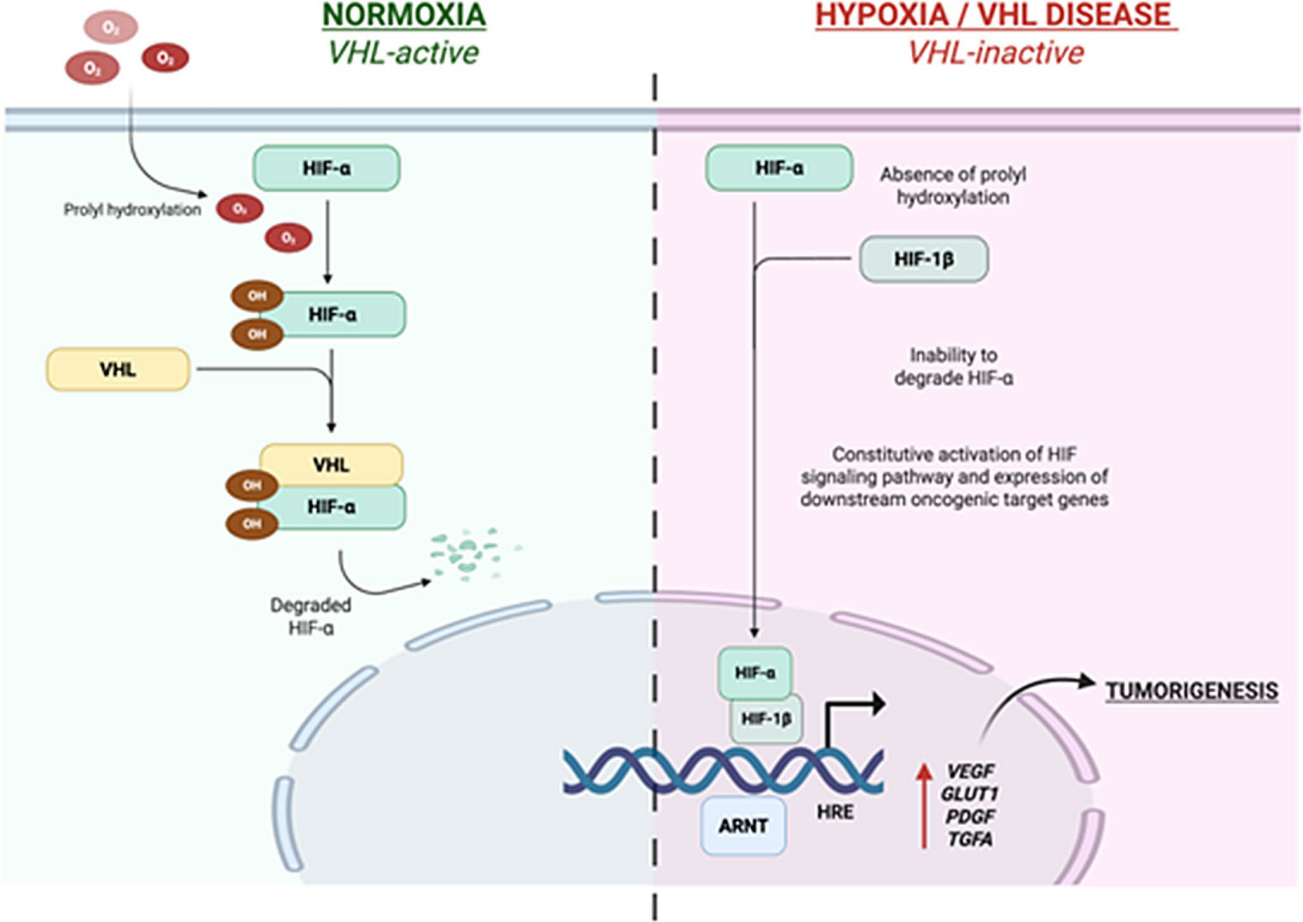
VHL/HIF signaling under normal/VHL-active and hypoxic/VHL disease/VHL-inactive conditions. under normal (normoxic) conditions, hydroxylated HIF-α (including both HIF-1α and HIF-2α) is bound by VHL and targeted for degradation by the proteosome. under hypoxic conditions and/or the setting of VHL-inactivation, HIF-α accumulates and associates with its partner HIF-1β and translocates to the nucleus, where the HIFα/HIFβ heterodimer binds to HREs, promoting transcription of HIF target genes that drive tumorigenesis. *Abbreviations:* HIF (hypoxia-inducible factor), VHL (von hippel-lindau), ARNT (aryl hydrocarbon receptor nuclear transporter gene), HRE (hypoxia-response elements), VEGR (vascular endothelial growth factor), PDGF (platelet-derived growth factor), GLUT1 (glucose transporter 1), TGFA (transforming growth factor alpha). This figure was created in Biorender

The VHL gene, a key HIF pathway regulator, is a tumor suppressor gene encoding two isoforms of VHL protein (pVHL). HIF, an oxygen-dependent transcription factor composed of an unstable oxygen-sensitive α-subunit (HIF-1α, HIF-2α, and HIF-3α) and a stable β-subunit (HIF-1β), regulates cellular adaptation in hypoxic microenvironments [12]. Under normoxic conditions, pVHL targets HIFα subunits, particularly HIF-2α, for ubiquitin-mediated proteasomal degradation, limiting tumorigenic gene expression programs (Fig. 1). In contrast, in hypoxic tumor microenvironments and/or in pVHL inactivation, HIF-2α accumulates, dimerizes with HIF-1β, and binds to hypoxia-response elements (HREs), leading to constitutive overexpression of the pro-oncogenic HIFα transcription factor, which has over 800 downstream target genes that can promote cell proliferation, metabolism, and angiogenesis (Fig. 1) [12, 13]. Constitutive HIF-2α activation from pVHL inactivation is a central driver of tumorigenesis in VHL disease [8, 12–14]. This led to the development of belzutifan, a second-generation, selective HIF-2α inhibitor.

Belzutifan disrupts HIF-2α/HIF-1β dimerization, suppressing downstream oncogenic transcription and tumorigenesis. In 2021, belzutifan received FDA approval following a phase 2 open-label, single-arm LITESPARK-004 trial that demonstrated durable treatment response across VHL-associated neoplasms—including renal cell carcinoma (RCC), pancreatic neuroendocrine tumors (PNETs), and CNS HBs [12, 13, 15, 16]. All had localized RCC and pancreatic lesions; 82% (*n* = 50) had measurable CNS HBs and 20% (*n* = 12) had retinal HBs; patients received oral belzutifan 120 mg/day.^15^ In CNS hemangioblastomas, the objective response rates (ORR) ranged from 44 to 76%, with a median disease control rate reaching 90% [12, 17].

Accumulating evidence supports the efficacy of belzutifan in VHL-associated hemangioblastomas, with encouraging response rates, disease control, and tolerability in recent prospective trials and case reports [11, 16–18]. However, belzutifan therapeutic efficacy in sporadic HBs (sHBs) lacking identifiable VHL alterations remains unexplored. To our knowledge, we present the first case of sustained tumor reduction in a sporadic CNS HB treated with belzutifan, suggesting that HIF-2α pathway dependence may be present even in the absence of detectable VHL genetic aberrations.

## Case description

**Fig. 2:**
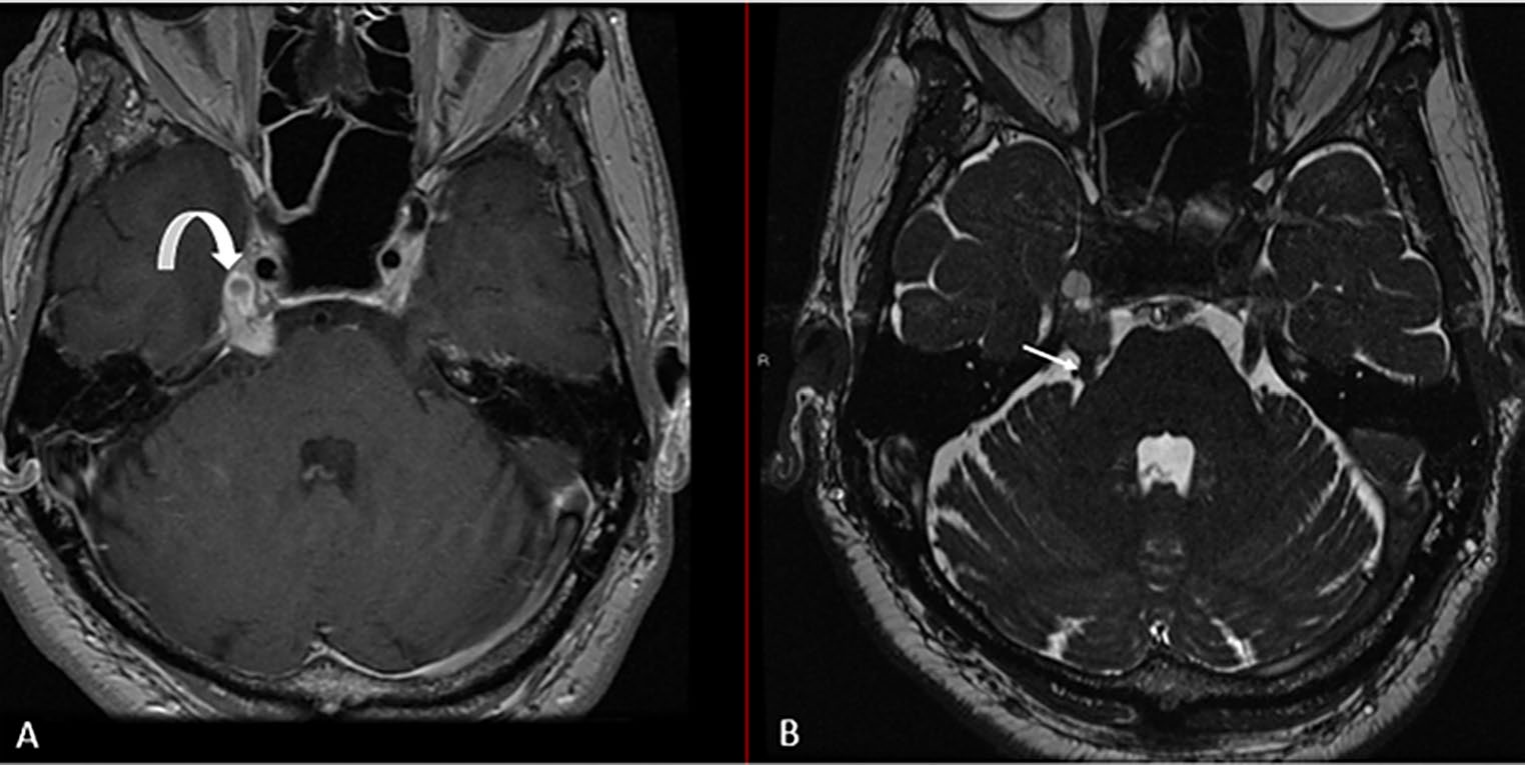
MRI brain axial T1 post-contrast (**A**) and axial T2 (**B**) show a heterogeneously enhancing mass involving the distal cisternal segment of the right trigeminal nerve (white arrow) and expanding the right Meckel’s cave; a non-enhancing cystic component is seen in Meckel’s cave (curved arrow)

A 65-year-old man initially presented in 2016 with subacute right-sided retro-orbital and occipital headaches, hemifacial numbness, and imbalance. A brain MRI (Fig. 2) revealed an extra-axial 1.2 × 1.0 cm heterogeneously enhancing mass, involving the distal cisternal segment of the right trigeminal nerve and expanding the right Meckel’s cave. He was diagnosed with presumed right trigeminal schwannoma and was treated with single-fraction CyberKnife radiosurgery (12.5 Gy) in August 2016. Serial MRIs demonstrated treatment response, with a slight volumetric reduction of the right trigeminal nerve mass, with the cisternal portion measuring 0.9 × 1.0 cm (previously 1.2 × 1.0 cm), and the Meckel’s cave extension measuring 1.1 × 0.6 cm (previously 1.3 × 0.8 cm).

From 2018 to 2021, he experienced progressive headaches, hemifacial neuralgia, blurred vision, episodic bilateral epiphora, and tinnitus, with interval locoregional tumor progression on MRI. In March 2021, the patient underwent a right subtemporal craniotomy with microsurgical tumor resection at an outside institution. The tumor was highly vascular and adherent to CN V, with intentionally residual disease in the cavernous sinus; other cranial nerves were uninvolved, and the brainstem-abutting component was completely resected. The postoperative course was complicated by a small right pontine infarct and transient left-sided weakness.

**Fig. 3:**
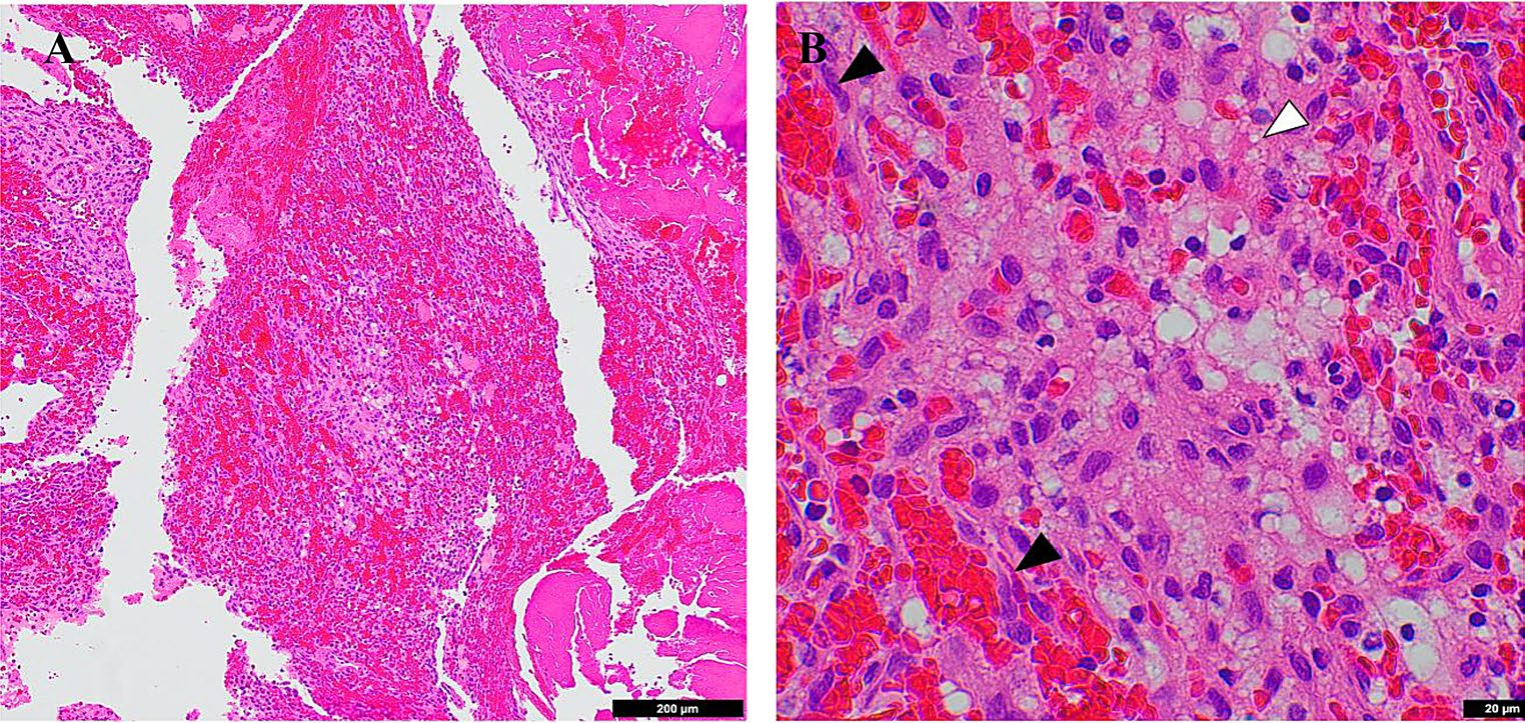
Tumor histopathology. Hematoxylin and eosin staining showing histological hallmarks of hemangioblastoma. (**A**) low-power view (10X; scale bar 200 µm) shows a cellular tumor with abundant hemorrhage, (**B**) high-power (60X; scale bar 20 µm) view shows networks of delicate capillaries (black arrowhead) and tumor cells with abundant cytoplasmic lipid droplets (white arrowhead)

Pathology demonstrated a well-circumscribed, highly cellular tumor composed of cells with finely vacuolated cytoplasm, along with numerous delicate capillaries and hemorrhage in the background (Fig. 3a–b). These findings were consistent with hemangioblastoma, CNS WHO grade 1. Subsequent peripheral blood testing using a multigene hereditary cancer panel (Ambry Genetics), including VHL analysis, did not reveal any pathogenic variants, variants of unknown significance, or copy number alterations. Tumor molecular sequencing was not performed.

Post-operative imaging initially showed stable residual disease until December 2021, when progressive tumor growth was noted within the Meckel’s cave/cavernous sinus, with extension into the cerebellopontine angle (CPA) and right middle cranial fossa. In March 2022, he underwent proton beam re-irradiation to the right skull base, receiving 50.6 Gy in 46 twice-daily fractions. Despite prior SRS, subtotal resection, and re-irradiation, the tumor progressed, accompanied by worsening chronic neurological deficits.

In February 2024, the patient pursued a second opinion at the University of Virginia (UVA) Medical Center to explore treatment options. His neurologic exam was notable for right hemifacial hypoesthesia, mild bilateral ptosis (right > left), right cranial nerve VII and XII paresis, right-sided hearing impairment, and subtle truncal ataxia. A review of serial brain MRIs (2016—2023) revealed substantial tumor progression involving the right Meckel’s cave and cavernous sinus and extension to locoregional supra-/infra-tentorial regions, with mass effect on the right mesial temporal lobe and midbrain. Treatment options were limited due to the lesion’s anatomical complexity and prior radiation. Repeat cytoreductive surgery was considered high risk, and the patient expressed a preference to avoid further surgery. Given limited local treatment options, systemic therapy with belzutifan was pursued, based on its efficacy in VHL-associated HBs and emerging evidence of VHL-HIF pathway dysregulation in sporadic HBs [19].

**Fig. 4:**
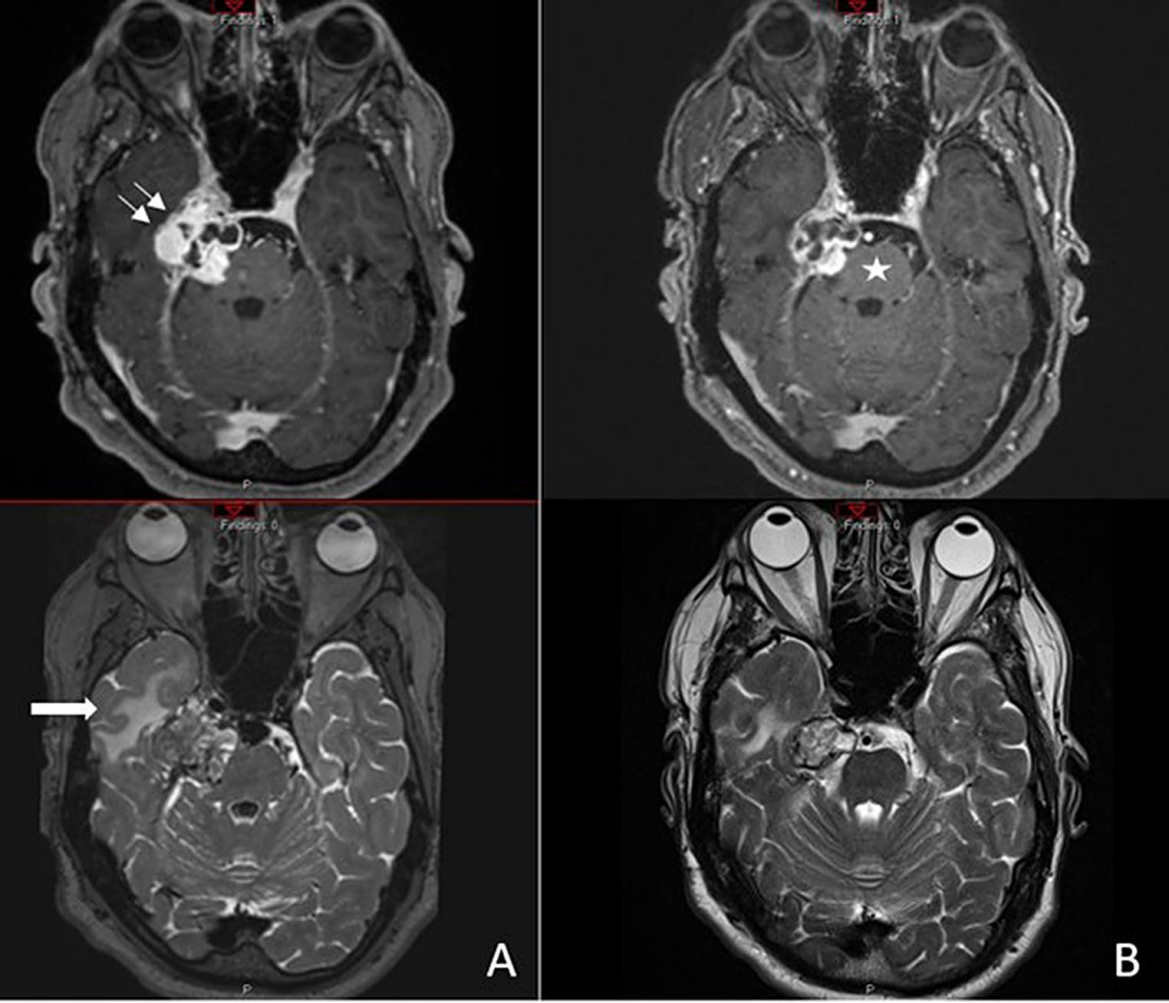
Brain MRI showing belzutifan-induced tumor reduction of sporadic hemangioblastoma of the right Meckel’s cave. (**A**) baseline MRI before starting belzutifan, (**B**) follow-up MRI after 9 months of belzutifan 80 mg daily treatment. (**A** and **B**) axial T1 post contrast (top) and axial T2 (bottom) images show: 1) a substantial decrease in the overall size of the lobulated, heterogeneously enhancing solid-cystic mass involving the right trigeminal nerve, the right cavernous sinus, and expanding the right Meckel’s cave (from 3.5 × 3.5 cm in A to 2.6 × 2.9 cm in **B**); 2) decreased size in the solid components (double white arrow); 3) decreased mass effect on the pons (white star) and improved right temporal edema (white arrow)

**Fig. 5:**
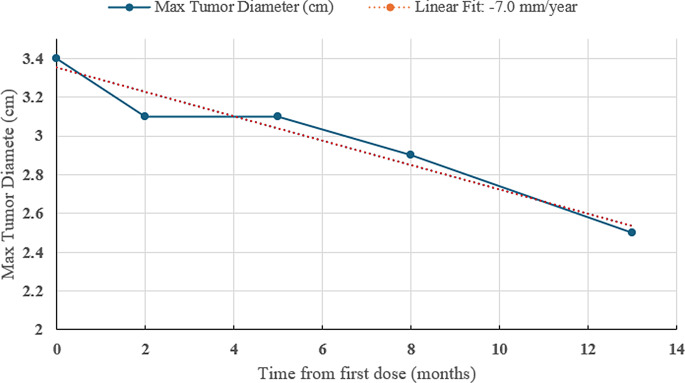
Calculated linear growth reduction from May 2024 through April 2025. The calculated linear fit equation was *Y = (−0.0021)X + 3.363*, with R^2^ = 0.9438, where *Y* represents tumor size (cm) and *X* represents time from first dose (months)

Belzutifan was initiated at 120 mg daily but required a brief dose interruption two weeks into treatment due to malaise, nausea, worsening right hemifacial neuralgia, and headaches; hemoglobin (Hb) and hematocrit (Htc) were within normal range. After approximately six weeks on full-dose belzutifan, he developed symptomatic fatigue and anemia, with a cumulative decline in Hb to 10.1 g/dL and in Htc to 29.7%. This prompted a second treatment interruption, followed by a dose reduction to 80 mg daily continued thereafter. Despite dose interruptions and reductions, MRI at month 2 demonstrated tumor regression and clinical stabilization. Serial MRIs at months 5, 9 (Fig. 4), and 13 showed sustained, gradual tumor reduction, corresponding to a median linear growth rate reduction of −7.0 mm/year in maximal tumor diameter (Fig. 5) [17].

Anemia and hypoxia are well-recognized adverse effects of belzutifan, warranting routine monitoring [15, 17, 20, 21]. Median SpO₂ values remained stable, ranging from 94 to 98% during treatment. As expected with HIF-2α inhibition and suppression of erythropoietin (EPO) transcription, the patient developed treatment-related anemia within the first 2 months of therapy, with Hb decreasing from 16.6 g/dL to 10.1 g/dL and Htc from 49.7% to 29.7%, respectively. Hemoglobin and hematocrit levels subsequently stabilized following a reduction in belzutifan dose to 80 mg daily. This is similar to the plateauing hematologic profile observed in clinical trials of belzutifan [15]. Treatment has remained ongoing with Hb > 10.0, Htc > 32.0%, and tolerable fatigue

Notably, this case demonstrates sustained tumor reduction while on dose-reduced belzutifan at 80 mg daily. Treatment was generally well tolerated, with grade 1–2 fatigue attributed to therapy and other grade 1–2 symptoms attributed to tumor-associated sequelae. This case demonstrates comparable safety and efficacy of belzutifan in sporadic CNS hemangioblastomas compared with VHL-associated CNS hemangioblastomas, highlighting the potential of HIF-2α inhibition as a therapeutic option in selected cases.

## Discussion

Hemangioblastomas, based on the 2021 WHO CNS classification, are mesenchymal, non-meningothelial WHO grade 1 vascular CNS neoplasms that are slow-growing and relatively rare, with an overall incidence of 0.141 per 100,000 person-years [2]. Approximately 75% of CNS hemangioblastomas are sporadic (sHBs), while 25–30% are associated with VHL [3, 19, 22]. Patients with VHL-associated HBs typically present earlier (20s to 30s) than sHBs (40s to 60s).^6,7,10,23^ sHBs rarely occur multiply, whereas 70–90% of patients with VHL disease will develop multiple HBs [2, 5, 23].

CNS HBs are the leading cause of mortality in patients with VHL disease. They are associated with significant morbidity through mass effect, progressive cyst formation, and/or hemorrhage, given their predilection for anatomically constrained regions (i.e. posterior fossa, Meckel’s cave) [5, 8, 17]. Historically, management relied on surgery and SRS, with limited systemic treatment options before belzutifan. These complexities necessitate a multidisciplinary approach for CNS HBs.

Based on initial MRI and operative findings, this patient’s tumor most likely originated in close association with the right trigeminal nerve, with extension into Meckel’s cave at presentation—an exceedingly rare localization. Excluding CPA and optic-chiasmatic cistern HBs, only four cases of trigeminal nerve/Meckel’s cave HB have been reported, to our knowledge [9, 10, 24, 25]. Among these cases, two were initially presumed schwannomas due to presentation as solitary, predominantly enhancing masses, with CNS HBs diagnosed only after resection following tumor progression [10, 25]. In the other two cases, CNS HB was considered at presentation, based on hypervascular features and/or cystic morphology, and one led to VHL disease diagnosis based on multifocal, characteristic lesions [9, 10, 24].

Of diagnostic importance, the majority of supratentorial (ST) hemangioblastomas reported in recent studies were solid (61–67%), solid-cystic (22%), and classic cystic lesions, with mural nodules representing only 10.6–11% of cases [6, 26, 27]. These findings suggest that “cranial nerve” hemangioblastomas (mesoderm-derived stromal cells, arising in leptomeninges in close association with cranial nerves) may be under-recognized due to radiographic mimicry of other common extra-axial tumors such as schwannomas and meningiomas—posing diagnostic and therapeutic challenges [24].

In this case, a symptomatic right trigeminal mass was initially diagnosed as a trigeminal schwannoma. Upon retrospective review of outside hospital neuroimaging (Fig. 2), we noted a non-enhancing cystic component within the solid enhancing nodule. These imaging features, had they been supplemented by growing knowledge of HB manifestations, might have led to consideration of supratentorial HB and prompted consideration of diagnostic surgical microdissection over first-line SRS. However, surgical resection of HBs involving the trigeminal nerve and Meckel’s cave is particularly challenging due to its anatomical continuity with the cavernous sinus/prepontine cistern, as well as its proximity to cranial base neurovascular structures.

A retrospective review of 237 cases of ST hemangioblastomas showed that STR and radiotherapy were inferior to GTR regarding PFS, though not feasible in high-risk regions and warranting consideration of alternative treatments in those cases [7]. Growing evidence has shown durable 5- and 10-year local control (LC) rates with SRS in VHL-associated (74% to 93.3%) and sporadic hemangioblastomas (61% to 80%) [7, 27]. Notably, these outcomes pertain to CNS HBs broadly, as data specific to cranial nerve HBs remain scarce due to their rarity. These findings underscore the importance of patient-tailored, multidisciplinary management in considering surgical feasibility, tumor phenotype, prior treatments, and alternative options—both at diagnosis and in the setting of disease progression/recurrence. In our case, despite SRS, subtotal resection, and re-irradiation, the tumor progressed, illustrating the limitations of local therapy and supporting the multidisciplinary decision to trial belzutifan.

In the LITESPARK-004 study, which included the assessment of 184 CNS HBs, belzutifan was administered at a dose of 120 mg/day.^17^ Solid CNS hemangioblastomas showed a higher responsiveness (76% ORR; *n* = 19) compared to solid-cystic lesions (44% ORR; *n* = 22), with median linear growth rate (LGR) decline to −1.6 mm/year for solid and −1.1 mm/year for solid-cystic lesions [15, 17]. The median time to response was 3.1 months in solid and 5.4 months in solid-cystic lesions. In this patient with a sporadic, non-VHL-associated solid-cystic CNS hemangioblastoma, the earliest radiographic treatment response occurred at 2.3 months of treatment, with approximately 8.8% reduction in maximal tumor diameter. Most notably, our patient demonstrated a median post-treatment LGR reduction of −7.0 mm/year at treatment month 13, exceeding the rates observed in VHL-associated cohorts. However, the irregular lobulated, solid-cystic morphology of our case’s tumor may introduce variability in measurement compared to the LITESPARK-004 trial’s Approach 1 methodology for assessing solid-cystic CNS hemangioblastomas, potentially overestimating the treatment-related volumetric response. Nonetheless, the consistent volumetric tumor reduction observed across serial MRIs over 13 months and on dose-reduced belzutifan supports a sustained antitumor effect of belzutifan in sporadic CNS hemangioblastoma.

The most common reported adverse effects of belzutifan include low-grade anemia (90%), fatigue (66%), headache (41%), and dizziness (39%), with no grade 4 or 5 treatment-related AEs [15, 17]. Similarly, our patient experienced treatment and dose-limiting anemia and fatigue, without any high-grade toxicities. Aligning with other belzutifan studies, his hemoglobin and hematocrit levels showed a peak decline within the first 2 months of treatment, consistent with EPO suppression related to HIF-2 inhibition, followed by stabilization after dose adjustment. Median SpO_2_ values remained within physiological range throughout treatment. These findings reinforce the relative tolerability and safety profile of belzutifan.

Sporadic HBs may remain susceptible to HIF-2 inhibition despite the absence of detectable pathogenic VHL alterations, as in our case. In VHL disease, tumorigenesis arises from HIF-driven oncogenic signaling secondary to biallelic VHL inactivation, following Knudson’s two-hit hypothesis. In sporadic ccRCC, somatic biallelic VHL inactivation has been observed in over 90% of cases [12]. In contrast, sporadic CNS HBs demonstrate lower rates of somatic biallelic inactivation, ranging from <10% to 47%, depending on the detection methods and study cohorts [12, 19]. Additionally, when present, somatic VHL mutations in hemangioblastomas are limited to the neoplastic stromal cells (10–20% of tumor cellularity), whereas vascular and recruited components lack these alterations [19, 28]. This cellular heterogeneity may contribute to the under-detection of a spectrum of cryptic VHL alterations using traditional tumor sequencing methodology [19]. Deep-coverage sequencing has improved sensitivity for detecting VHL alterations in sporadic HBs, as demonstrated in a 2014 study that identified single-allele VHL inactivating alterations in 78% of cases and biallelic VHL inactivation in 47%—a substantially higher rate than prior reports [19].

In our case, serum testing did not identify a pathogenic VHL variant; however, the tumor could still theoretically harbor undetected low-frequency or tumor-restricted alterations affecting the VHL-HIF pathway, representing a plausible point of vulnerability to belzutifan. Whole-genome or deep targeted tumor sequencing could potentially identify these molecular alterations. However, given accumulating evidence that dysregulated VHL-HIF pathway signaling is a central pathogenic mechanism in hemangioblastomas, belzutifan was initiated without further molecular characterization. Alternatively, this tumor’s susceptibility to belzutifan could stem from mutations affecting key VHL binding partners and leading to the same phenotype of dysregulated HIF signaling/accumulated HIF-2α [19]. One possible candidate gene mutation described is *TCEB1*, which encodes elongin C, a critical VHL binding partner whose mutation may similarly disrupt HIF regulation, rendering sHBs susceptible to HIF-2α inhibition [19, 29]. These evolving insights into sHB’s etiopathogenesis provide a compelling rationale for the potential efficacy of belzutifan in sHB.

Limitations of this case include the single-patient design and lack of molecular confirmation of somatic VHL status. Also, the durability of the response beyond the 13-month analysis remains unknown. Notably, across studies, the duration of belzutifan treatment—even in VHL-associated neoplasms—has yet to be established. Nonetheless, this case provides encouraging preliminary evidence to support further investigation of HIF-2α inhibition in larger cohorts of sporadic CNS hemangioblastomas.

## Conclusion

We present the first reported case of belzutifan-induced tumor regression in a sporadic, non-VHL-associated hemangioblastoma. This case supports the concept that functional impairment of the VHL-HIF pathway may be present in sporadic HBs through mechanisms not captured by standard germline testing and/or conventional tumor sequencing, including cryptic mutations, low-allele-frequency alterations confined to stromal cells, or disruption in VHL protein function. Additionally, belzutifan targets downstream HIF-2 signaling, providing a therapeutic rationale that may not depend on identifying a pathogenic VHL alteration.

The findings in the current case support exploration of belzutifan beyond its current limited indication for VHL-associated HBs. We recommend considering belzutifan as a potential systemic option in select cases of progressive CNS sHBs for which surgical and/or radiotherapeutic approaches are limited. Further studies of belzutifan in larger cohorts of CNS sHBs are warranted.

## Data Availability

No datasets were generated or analysed during the current study.
